# Editorial: Novel Composites and Multi-Material Assembly Approaches for Tissue Regeneration

**DOI:** 10.3389/fbioe.2020.00680

**Published:** 2020-07-14

**Authors:** Ravi Sinha, Fergal J. O'Brien, Sandra Camarero-Espinosa

**Affiliations:** ^1^Maastricht University, Maastricht, Netherlands; ^2^Royal College of Surgeons in Ireland, Dublin, Ireland

**Keywords:** composites, tissue engineering, biofabrication, multi-material, regenerative medicine

In this Research Topic entitled “Novel composites and multi-material assembly approaches for Tissue Regeneration,” we aimed to bring forth new insights and ideas from our peers who combine various materials to create complex tissue-regeneration solutions. As we wrap up this topic for now, we are happy to report that we were able to attract several interesting research studies, which generated significant new insight for our field. On composite materials, we received studies combining collagen composites with other biological factors, native extracellular matrix composites with hydrogels and cellulose-hydrogel composites among biologically sourced materials; and polymer-drug, polymer-polymer and polymer-hydrogel composites among (mostly) synthetic materials. On multi-material fabrication approaches, the papers received covered pressure based extrusion (bioprinting), composite material electrospnning, and an automated sheet rolling technique to obtain 3D constructs from electrospun films.

To highlight some of the interesting findings from this Research Topic, de Pinho et al. found a simple way to improve the wettability of electrospun polymeric scaffolds (which is important for interactions with cells) by creating a composite with alginate which could later form a hydrogel. This was done by adding alginate to the polymer solutions before the electrospinning process.

Frost et al. used cellulose nanocrystals (CNC) to increase the viscosity of light cross linkable PEGDA hydrogels enough to achieve extrusion based bioprinting, which was otherwise not possible. Their CNC-PEGDA composites showed an improvement in mechanical properties as well over the PEGDA gels alone.

While the former two studies focused on novel materials and fabrication methods, do Amaral et al. delved deeper into biological aspects to confirm that supplementing of clinically-relevant collagen-glycosaminoglycan scaffolds with platelet-rich plasma (PRP) gels provided a composite material with improved ability to attract blood vessels while maintaining a beneficial immune response.

Shamsah et al. produced poly(ε-caprolactone) (PCL) and poly(L-lactic) acid (PLLA) composite electrospun fibers which had improved load bearing ability over PCL alone and then they rolled sheets of aligned fibers and cells into tubes, using an automated device, which allowed them to build 3D structures layer by layer and resembling the *in vivo* tissue architecture of annulus fibrosus.

Shridhar et al. removed cells from bone and adipose tissue and created composites between these cell-free extracellular matrices (ECM) and methacrylated chondritin sulfate hydrogels to study how tissue specific ECM can affect the differentiation of adipose derived stem cells. They show that tissue specific ECM can act synergistically with soluble factors to improve stem cell differentiation toward the tissue from which the ECM was sourced.

O'Leary et al. loaded a drug (all-trans retinoic acid) in electrospun chitosan coated PCL scaffolds and showed using *in vitro* studies how the controlled release of the drug enhanced its potential for tracheal tissue regeneration.

Thus, the topic ended up reflecting the wide diversity of this field of composite biomaterials, including composites to improve physical properties (de Pinho et al.; Frost et al.; Shamsah et al.), affecting cell behavior (do Amaral et al.; Shridhar et al.; O'Leary et al.), or enabling controlled fabrication of scaffolds (Frost et al.). Besides all papers studying material combinations with few precedents, the gradient bioprinting demonstrated by Frost et al. and the controlled alignment between layers used by Shamsah et al. indicate that the field is heading toward better control on the distribution and interaction between the materials in order to get the best out of these novel composite materials.

Finally, we (topic editors) would like to thank all the authors, guest editors and reviewers for their valuable contributions to this Research Topic.

## Author Contributions

RS prepared the initial draft. FO'B and SC-E reviewed and added inputs. All authors contributed to the article and approved the submitted version.

## Conflict of Interest

The authors declare that the research was conducted in the absence of any commercial or financial relationships that could be construed as a potential conflict of interest.

